# Supplementary material for: A fault-intrusion-tolerant system and deadline-aware algorithm for scheduling scientific workflow in the cloud
Source: PeerJ Comput Sci. 2021 Nov 2;7:e747. doi: 10.7717/peerj-cs.747 (PMC8576554; doi:10.7717/peerj-cs.747)
Supplement: Supplemental Information 6 [file peerj-cs-07-747-s006.zip › Myproject1.1/docs/org/cloudbus/cloudsim/util/WorkloadModel.html]

WorkloadModel


JavaScript is disabled on your browser.


- Overview
- Package
- Class
- Use
- Tree
- Deprecated
- Index
- Help

- Prev Class
- Next Class

- Frames
- No Frames

- All Classes

- Summary:
- Nested |
- Field |
- Constr |
- Method

- Detail:
- Field |
- Constr |
- Method


org.cloudbus.cloudsim.util

## Interface WorkloadModel

- All Known Implementing Classes:
  :   WorkloadFileReader

  ---

    

  ```
  public interface WorkloadModel
  ```

  This interface defines what a workload model should provide. A workload model generates a list of
  jobs that can be dispatched to a resource by `Workload`.

  Since:
  :   5.0

  See Also:
  :   `Workload`,
      `WorkloadFileReader`

- - ### Method Summary

    Methods

    | Modifier and Type | Method and Description |
    | --- | --- |
    | `java.util.List<Cloudlet>` | `generateWorkload()` Returns a list with the jobs generated by the workload. |

- - ### Method Detail


    - #### generateWorkload

      ```
      java.util.List<Cloudlet> generateWorkload()
      ```

      Returns a list with the jobs generated by the workload.

      Returns:
      :   a list with the jobs generated by the workload.


- Overview
- Package
- Class
- Use
- Tree
- Deprecated
- Index
- Help

- Prev Class
- Next Class

- Frames
- No Frames

- All Classes

- Summary:
- Nested |
- Field |
- Constr |
- Method

- Detail:
- Field |
- Constr |
- Method
